# Resilience through sports: Ukraine and Latvia confront geopolitical challenges

**DOI:** 10.3389/fspor.2025.1563761

**Published:** 2025-06-09

**Authors:** Marina Kamenecka-Usova, Maxym Tkalych

**Affiliations:** ^1^ Social Sciences Faculty, Rīga Stradiņš University, Riga, Latvia; ^2^ Law Department, EKA University of Applied Sciences, Riga, Latvia; ^3^ Civil Law Department, Zaporizhzhia National University, Zaporizhzhia, Ukraine

**Keywords:** sports sector policy, national security in sports, Ukraine and Latvia collaboration, resilience in sports, sports and politics

## Abstract

This perspective article examines the response of the sports sector in Ukraine and Latvia to contemporary geopolitical challenges, particularly those arising from ongoing war in Ukraine. As tensions escalate, both nations have taken significant measures within the sports sector, adopting new legal frameworks and policies to align with national security goals. By analyzing recent legislative changes, sanctions, and collaborative efforts within international sports organizations, this article explores how Latvia and Ukraine's sports sectors are addressing political challenges. These efforts reflect a growing recognition of the need to uphold ethical standards, protect athletes, and use sports as a platform for resilience in the face of geopolitical threats.

## Introduction

The answer to the question of what connects world tennis star Novak Djokovic and the 20th-century postage stamps is neither simple nor immediately apparent. However, it is explored in the recent intriguing works of Eggeling (1) and Lebed and Morgulev (2). Eggeling examines the interplay of politics and power within the global tennis circuit, while Morgulev and Lebed highlights how authoritarian regimes used sports, vividly portrayed through postage stamps, as a platform for showcasing their superiority and disseminating government propaganda.

The relationship between sports and politics has been extensively examined by renowned scholars such as Grix (3), Bertoli (4), Murray (5), Pamment (6), Dichter (7), Pigman (8) and others. Real-world examples like Ping Pong Diplomacy, Cricket Diplomacy, the “La Guerra del Futbol,” and tragedies such as at the 1972 Munich Olympics underscore the enduring relevance of this subject over the years.

Recent years of our lives have been no exception as the sports sector has increasingly found itself at the crossroads of geopolitics and national security concerns. This article demonstrates the intersection between sports, war, and politics in nowadays Eastern Europe, highlighting the sports sector's response, including reforms in sports legislation to align with broader political priorities. In Ukraine and Latvia, this intersection has necessitated a shift in how sports organizations, legal institutions, and policymakers navigate the impact of hostilities on sports. Both countries, sharing a common history as former Soviet republics and having been exposed to similar geopolitical risks, have demonstrated that sports are not merely a platform for athletic excellence but also a significant avenue for national expression and integrity preservation in response to geopolitical pressures.

While Ukraine's motivations and reasons are widely understood by the international community, Latvia's actions require a closer look at its geographic location—specifically its proximity to Russia—its history as a former Soviet republic (de facto one of the Soviet Union's constituent republics from 1940 to 1941 and again from 1944 to 1990), and its population demographics, with approximately 30%–35% of permanent residents speaking Russian as their mother tongue, including 180,455 (9) non-citizens (i.e., “nepilsoņi”) as of early 2024 (10).

However, it's essential to recognize that ongoing geopolitical situation not only threatens the integrity of Ukraine, its territorial borders, and its population but also poses a significant risk, as with any major shock to the nation, of hindering the progress of sports and delaying athletic achievements for many years. Understanding these challenges can offer the international sports community valuable insights into ways to support Ukrainian sports, helping to minimize the risks and mitigate the consequences. In light of this, we outline the perspective of the challenges, responses, and potential avenues for assistance.

### Latvia’s responses to geopolitical pressures

In March 2022, just one month after Russia invaded Ukraine, a survey of permanent residents of Latvia aged 18–74 revealed a prevailing sentiment that holds Russia accountable for the conflict. A majority (57%) of respondents identified Vladimir Putin as responsible for the war in Ukraine, with an additional 11% attributing blame to the Russian state. Further analysis of survey responses across socio-demographic groups showed that Latvians, particularly those under 24, were more likely (over 70%) to hold Putin personally responsible.

In contrast, 17% of Latvian respondents, and a notable 42% of Russian-speaking respondents, identified the U.S. and NATO as responsible for the conflict. Ukraine itself was cited as the instigator by 4% of respondents, while 6% believed that all involved parties shared responsibility for the war. The survey also revealed divisions within the population concerning support for Russia's actions, with 8% of participants (including 19% of Russian-speaking respondents) expressing support for President Putin's invasion of Ukraine. Nonetheless, a substantial majority of Latvian respondents (78%) opposed Russia's actions (11).

A closer look at regional responses highlights the unique perspectives within Latgale, where attitudes differed significantly from other Latvian statistical regions. In Latgale, only 39% of respondents considered Russia responsible, compared to over half in other regions. Moreover, a substantial number in Latgale attributed responsibility to the West, while both Latgale and Riga had a considerable proportion of respondents who were undecided on the matter (12, 13).

The survey results also underscored a sense of political alienation among Latvian residents who primarily speak Russian within their families—a phenomenon that has developed over decades and has been widely studied in Latvian academia. Dr. Ilga Apine has suggested that for much of this Russian-speaking population, Latvian culture and history remain distant, with identity often rooted in Soviet and Russian values. This notion is supported by 2022 survey data, which show that nostalgia for the past persists among Russian-speaking families, with strong affiliations to both modern Russia (20%) and, to an even greater extent, the former USSR (29%). This indicates that their connection to Russia is often grounded in historical identity rather than current political allegiance. Despite this, a significant 88% of Russian-speaking residents expressed a sense of belonging to Latvia (14).

The diversity of perspectives and lingering attachments to Russian identity among segments of the population likely contributed to the Latvian legislature's recent amendments to the sports law. Latvia's response to geopolitical challenges, particularly in light of Russia's invasion of Ukraine, includes decisive legislative action in the sports sector. On 28 April 2022—just two months after the invasion—the Saeima of the Republic of Latvia adopted amendments (No. 2022/83A.1) to the Sports Law, which came into force on 30 April 2022 and were reinforced in 2024. These amendments restrict Latvian athletes and teams from participating in events organized by Russia and Belarus and prohibit collaboration with sports agents and officials from these countries who may have ties to foreign intelligence services.

Further amendments to Article 10.1 of the Sports Law specify that members of the executive body of a sports federation must be Latvian citizens. Individuals who are staff members, employees, or informers of foreign state security, intelligence, or counterintelligence services, or the State Security Committee of the Latvian SSR (KGB), are prohibited from holding such positions. Candidates for executive roles must certify in writing that they are not subject to these restrictions and, within three months of election, provide a certificate from a competent public authority confirming their eligibility. Certificates from the Centre for the Documentation of the Consequences of Totalitarianism are not required for individuals born after August 21, 1991.

These amendments aim to prevent individuals potentially disloyal to the Latvian state and its Constitution from leading sports federations. They align with the ECHR ruling (*Anchev v. Bulgaria*, January 11, 2018) (15), which affirmed states’ broad discretion to implement measures protecting national security and addressing the legacies of non-democratic regimes. Accordingly, the law excludes individuals with ties to the USSR or Latvian SSR security services (16).

This strategic response reflects Latvia's determination to protect its athletes and sports sector from potential political influence while standing in solidarity with Ukraine. Such measures also underscore the Latvian government's commitment to safeguarding national integrity in the sports industry by limiting interactions with entities from countries perceived as aggressive or politically destabilizing. Given the complex social dynamics and varied public opinion revealed by the survey, the amendments can be seen as a strategic effort to align Latvia's sports policies with its stance on national security and geopolitical integrity.

The proactive and unified stance of the Baltic States is clearly demonstrated in the Joint Statement by the Baltic Foreign Ministers on Russian and Belarusian Participation in the 2024 Olympics (17). This declaration of Latvia, Estonia and Lithuania highlights key concerns and arguments about the potential inclusion of Russian and Belarusian athletes in the 2024 Paris Olympics under “neutral” status.

Key arguments were:

*Condemnation of Russia and Belarus*. The statement emphasizes that Russia, with Belarus as an accomplice, is waging an illegal war of aggression against Ukraine, aiming to erase a sovereign nation and Olympic participant. The ministers argue that the escalation of atrocities by Russia makes it unjustifiable to ease sanctions on Russian and Belarusian athletes.

*Opposition to “neutral” athlete participation*. Allowing athletes from Russia and Belarus to compete under neutrality is problematic because many of them receive direct state support from their regimes. Russia has historically exploited the presence of “neutral” athletes in international events for propaganda purposes, including promoting its military and recruitment efforts.

*Impact on Ukrainian sports*. The war has resulted in the deaths of hundreds of Ukrainian athletes and the deliberate destruction of sports infrastructure in Ukraine. Ukrainian athletes face immense challenges due to the war, making the idea of competing alongside Russian and Belarusian athletes unjust and morally unacceptable.

*Violation of Olympic principles*. The document asserts that Russia's actions violate core Olympic principles, including peace and humanity, as well as the United Nations Charter. It calls on the IOC to uphold its own restrictive measures and not compromise the integrity of the Olympic Games.

*Call for action*. The ministers urge the IOC to maintain the current sanctions and bar Russian and Belarusian athletes from participating in any capacity. They emphasize the IOC's responsibility to protect the values of sportsmanship and solidarity, supporting Ukraine and the global sports community against aggression.

In conclusion, the statement underscores the Baltic states’ commitment to supporting Ukraine and stresses that allowing Russian and Belarusian athletes into the 2024 Olympics would undermine the Olympic movement and embolden the use of aggression as a political tool. This document serves as a pivotal argument in international discourse regarding the intersection of sports, politics, and global conflict.

### The impact of Russia’s aggression on sports in Ukraine and Ukraine’s strategic response

Russia's military aggression against Ukraine since 2014, escalating to a full-scale invasion in 2022, has reshaped global politics and significantly disrupted the sports sector. Ukraine recognizes sports as a crucial platform for political influence and has leveraged this to isolate Russia and Belarus internationally. These efforts align with broader sanctions, demonstrating the increasing importance of sports diplomacy in conflict resolution and advocacy. As a dynamic tool for building bridges, strengthening international ties, and promoting national interests, sports diplomacy plays a pivotal role on the global stage—a notion examined in detail by Postlethwaite, Jenkin, and Sherry in their integrative review (18).

Key features of Ukraine’s sports policy during the war are:

#### Boycott and isolation

Ukraine has actively pursued the exclusion of Russian and Belarusian athletes and teams from the global sports community. This strategy is reflected in the International Olympic Committee's (IOC) sanctions from 2022 (19), which include:

• A ban on hosting international sports events in Russia and Belarus.
• Prohibition of Russian and Belarusian flags, anthems, and symbols at international competitions.
• Revocation of Olympic honors awarded to Russian political figures.

#### Political messaging through sports

Sports have become a platform for denouncing Russian aggression. Institutions like the Ministry of Youth and Sports, the National Olympic Committee (NOC) of Ukraine, and national sports federations actively advocate for the exclusion of Russia and Belarus from international sports. In particular, the Ministry of Youth and Sports of Ukraine, the NOC, and the Wrestling Federation appealed to the IOC with a demand to suspend Russian and Belarusian athletes from participating in the 2024 Olympic Games in Paris (20).

#### Ban on joint competitions with Russian athletes

Ukraine enforces a strict ban on competing alongside Russian and Belarusian athletes to prevent any legitimization of the aggressor states. The Resolution of the Ministry of Youth and Sports of Ukraine «On some issues of participation of official delegations of national teams of Ukraine in international competitions in Olympic, non-Olympic sports and sports for people with disabilities» № 2031 adopted on April 12, 2023 (21) prohibits Ukrainian national teams from participating in competitions involving athletes from Russia or Belarus.

#### Counteracting Russian propaganda

Ukraine collaborates with international federations and advocacy organizations to expose Russia's use of sports as a propaganda tool, ensuring that the global community is aware of its politicization of sports. «Russia and Belarus should not be able to use the success of their teams and athletes for propaganda purposes to legitimize their illegal invasion of Ukraine»—this was stated in a video address during the international forum «The Power of Sports Diplomacy» by the Minister of Sport, Tourism, Civil Society and Youth of the United Kingdom, Stephanie Peacock (22).

#### International sports diplomacy

Ukraine is at the forefront of efforts to sustain and expand sanctions against Russian and Belarusian sports bodies. These efforts reinforce Olympic principles of peace and justice, ensuring solidarity with nations opposing aggression. Among other things, during the work of the UN General Assembly in November 2024, the Ukrainian delegation stated that «We call on the international community to take a tough stance and continue to isolate Russia and Belarus from world sport as long as Russia's aggressive war against Ukraine continues» (23).

**Figure 1:**
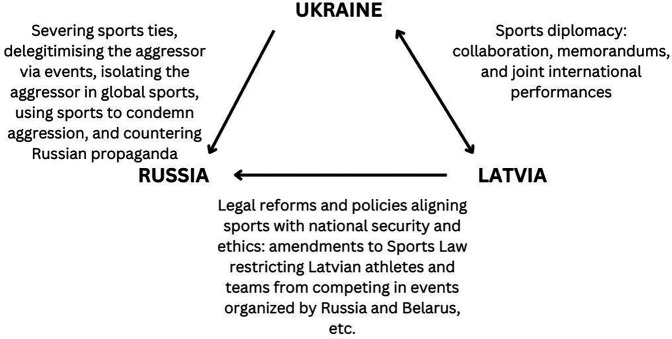
The responses of the sports sectors in Ukraine and Latvia to geopolitical challenges.

Figure 1 provides a visual overview of how the sports sectors in Ukraine and Latvia have responded to geopolitical challenges.

### Broader impact of war on the sports sector in Ukraine

The ongoing war in Ukraine has profoundly affected the sports sector, particularly as many athletes, coaches, and sports representatives have mobilized to support the Armed Forces or engage in volunteer efforts. Notable figures, such as legendary football coach Yuriy Vernydub (24), have left their coaching positions to defend their country, while athletes have organized charity events to raise funds for the military. This mobilization reflects a broader commitment within the sports community to contribute to national efforts during this crisis. Ukrainian football has faced immense challenges due to the war. Many clubs have experienced displacement; for instance, FC Mariupol (25) and FC Desna Chernihiv (26) lost their infrastructure as a result of hostilities. Additionally, clubs like Shakhtar Donetsk saw a significant loss of foreign players (27) but continued to compete in the Champions League, using these matches as platforms to raise awareness about the ongoing conflict. Since the beginning of the war, most Ukrainian sports clubs which were not closed have had no other choice but to shift their focus toward developing homegrown talent, demonstrating resilience in the face of adversity.

It is essential to emphasize that international support has become a crucial lifeline for Ukrainian sports. Organizations such as La Liga have stepped in to provide assistance, focusing on youth football development and offering financial support to help sustain operations amidst the turmoil (28).

Conversely, Ukrainian athletes who remained in Russia faced public condemnation; those who chose not to return were excluded from national teams and lost public backing, highlighting the complex repercussions of the conflict on individual careers.

As competitions resumed, the Ukrainian Premier League implemented strict safety measures, including matches played without spectators. These events have transformed into platforms for volunteer initiatives and support for those affected by the war. A particularly poignant example of this impact is the Donbas Arena, once a symbol of modern Ukrainian football (29). Located in Donetsk and regarded as Europe's first stadium built to UEFA Elite standards, it has been under occupation since 2014. The arena now stands as a stark reminder of the cultural and infrastructural losses inflicted by the conflict; previously hosting FC Shakhtar Donetsk matches and international events, its current state reflects the broader devastation experienced throughout eastern Ukraine. Overall, the war's impact on Ukraine's sports sector illustrates not only the immediate challenges faced by athletes and clubs but also underscores a resilient spirit within the community as they adapt to an unprecedented situation while striving to maintain their cultural identity amidst adversity.

### Future directions and diplomatic pathways

#### Strengthening international advocacy

Ukraine must enhance its partnerships with international sports federations to foster solidarity against aggressor states and secure support for its athletes. A noteworthy example is the collaboration between the Latvian Football Federation (LFF) and the Ukrainian Association of Football (UAF), which facilitates joint technical activities, educational initiatives, and opportunities for young Ukrainian players. This partnership also organizes summer camps in Latvia to support the recovery of Ukrainian children affected by the war.

#### Rehabilitation and development

Key priorities include providing robust support systems for athletes impacted by the war and creating pathways to integrate veterans into sports, fostering their physical and emotional rehabilitation.

#### Institutionalizing sports diplomacy

Ukraine has the potential to drive reforms within international sports organizations, advocating for ethical governance and policies that reject aggression.

#### Long-term exclusion strategies

Maintaining the exclusion of Russian and Belarusian athletes demands sustained advocacy and governance reforms that promote accountability and uphold international sports ethics.

Ukraine's proactive sports diplomacy underscores its resolve to isolate aggressor states while uniting the global community in solidarity. By employing a combination of legislative measures, public diplomacy, and international collaboration, Ukraine has positioned itself as a leader in using sports as a platform for geopolitical advocacy. Looking ahead, future initiatives could include developing secure frameworks for international collaborations, protecting athletes in politically sensitive environments, and establishing bilateral agreements that reinforce ethical sports participation. For both Latvia and Ukraine, such measures highlight the role of sports as a resilient force against geopolitical challenges.

Both nations have shown that sports can act as a powerful diplomatic tool and a reflection of national identity. Through decisive legal measures and international collaboration, they have effectively protected their sports sectors from external interference while presenting a united front on the global stage. A remarkable display of this unity through sports was seen when Latvia's top tennis player, Aļona Ostapenko, and her Ukrainian partner, Lyudmyla Kichenok, triumphed in the women's doubles final at the US Open Championship on September 6, 2024. The pair claimed their first-ever Grand Slam title together, showcasing the strength of their partnership.

As international sports increasingly intersect with geopolitical tensions, policymakers and sports organizations must adapt by prioritizing athlete protections, ethical governance, and robust partnerships. By doing so, Latvia and Ukraine can ensure their sports sectors remain symbols of integrity, unity, and resistance in a politically charged world.

## Data Availability

Publicly available datasets were analyzed in this study. This data can be found here: Oficiālās statistikas portals (2024) Nationality of the population: Latvian citizens, Latvian non-citizens (Latvia). https://stat.gov.lv/lv/statistikas-temas/iedzivotaji/iedzivotaju-skaits/kartes/k280-iedzivotaju-valstiska-piederiba.
